# An ensemble of structures of *Burkholderia pseudomallei* 2,3-bisphosphoglycerate-dependent phosphoglycerate mutase

**DOI:** 10.1107/S1744309111030405

**Published:** 2011-08-13

**Authors:** Douglas R. Davies, Bart L. Staker, Jan A. Abendroth, Thomas E. Edwards, Robert Hartley, Jess Leonard, Hidong Kim, Amanda L. Rychel, Stephen N. Hewitt, Peter J. Myler, Lance J. Stewart

**Affiliations:** a Seattle Structural Genomics Center for Infectious Disease (http://www.ssgcid.org), USA; b Emerald BioStructures Inc., 7869 NE Day Road West, Bainbridge Island, WA 98110, USA; c Department of Biology, University of Washington, Seattle, WA 98195-1800, USA; d Seattle Biomedical Research Institute, 307 Westlake Avenue North, Suite 500, Seattle, WA 98109, USA; e Departments of Global Health, Medical Education and Biomedical Informatics, School of Medicine, University of Washington, Box 357230, Seattle, WA 98195, USA

**Keywords:** *Burkholderia pseudomallei*, melioidosis, 2,3-bisphosphoglycerate-dependent phosphoglycerate mutase, fragment screening, vanadate, transition-state mimics, SSGCID, FBDD

## Abstract

*Burkholderia pseudomallei* is a soil-dwelling bacterium endemic to Southeast Asia and Northern Australia. *Burkholderia* is responsible for melioidosis, a serious infection of the skin. The enzyme 2,3-bisphosphoglycerate-dependent phosphoglycerate mutase (PGAM) catalyzes the interconversion of 3-phosphoglycerate and 2-phosphoglycerate, a key step in the glycolytic pathway. As such it is an extensively studied enzyme and X-ray crystal structures of PGAM enzymes from multiple species have been elucidated. Vanadate is a phosphate mimic that is a powerful tool for studying enzymatic mechanisms in phosphoryl-transfer enzymes such as phosphoglycerate mutase. However, to date no X-ray crystal structures of phosphoglycerate mutase have been solved with vanadate acting as a substrate mimic. Here, two vanadate complexes together with an ensemble of substrate and fragment-bound structures that provide a com­prehensive picture of the function of the *Burkholderia* enzyme are reported.

## Abstract 2

An ensemble of crystal structures are reported for 2,3-bisphosphoglycerate-dependent phosphoglycerate mutase from *B. pseudomallei*. The structures include two vanadate complexes, revealing the structure of a close analogue of the transition state for phosphate transfer.

## 1. Introduction

The phosphoglycerate mutase family encompasses enzymes that catalyse the interconversion of 3-phosphoglycerate and 2-phospho­glycerate. Most organisms utilize 2,3-bisphosphoglycerate-dependent phosphoglycerate mutase (PGAM; EC 5.4.2.1) to accomplish this reaction in glycolysis. In mammalian erythrocytes bisphospho­glycerate mutase (BPGM; EC 5.4.2.4) is the predominant form. PGAM converts 3-phosphoglycerate to 2-phosphoglycerate through a ping-pong mechanism in which a phosphoenzyme transfers a phosphate group from an active-site histidine to 3-phosphoglycerate to form a 2,3-bisphosphoglycerate intermediate that subsequently transfers the phosphate from the 3-position to yield the 2-phospho­glycerate product and a reconstituted phosphoenzyme. The primary reaction catalysed by BPGM is the conversion of 1,3-bisphospho­glycerate to 2,3-bisphosphoglycerate. The mechanism of BPGM begins with an unphosphorylated enzyme, the active-site histidine of which performs a nucleophilic S_N_2 attack on the 1,3-bisphospho­glycerate substrate to produce phosphohistidine and 3-phospho­glycerate. The 2′-hydroxyl group then performs a second nucleophilic attack that transfers the phosphate from the active-site histidine to the substrate, forming 2,3-bisphosphoglycerate. PGAM and BPGM enzymes typically share ∼50% sequence identity and highly con­served protein folds and readily catalyse each other’s primary reactions, albeit with decreased efficiency.

Commensurate with its importance as a key enzyme in a primary metabolic pathway, PGAM has been the subject of extensive investigation by X-ray crystallography. Three-dimensional crystal structures of PGAM have been solved from Gram-negative bacteria such as *Escherichia coli* (Bond *et al.*, 2002 ▶) and *Thermus thermophilus* (PDB entry 1v37; M. Sugahara, S. Yokoyama, S. Kuramitsu, M. Miyano, T. Iizuka & T. Kunishima, unpublished work), from the Gram-positive pathogen *Mycobacterium tuberculosis* (Müller *et al.*, 2005 ▶) and from *Saccharomyces cerevisiae* (Crowhurst *et al.*, 1999 ▶), to name a few. Here, we present six X-ray crystal structures of an additional Gram-negative bacterial PGAM from *Burkholderia pseudomallei* bound to various ligands.

*B. pseudomallei* is a soil-dwelling bacterium endemic to Southeast Asia and Northern Australia. *Burkholderia* is responsible for melioidosis, a serious infection of the skin. As a key enzyme in glycolysis and energy metabolism, PGAM is a potential target for novel antibiotics. To this end, several X-ray crystal structures with various ligands were solved for *Burkholderia* PGAM. While single-crystal structures of important enzymes are informative, the most valuable structural insights can be gleaned from ensembles of structures, which allow researchers to infer reaction mechanisms and may even provide a basis for the rational design of small-molecule inhibitors.

An important set of tools in the structural biologist’s repertoire are the transition-state analogs. These are small molecules whose unique chemistries allow the crystallographer to capture and visualize structures that approximate the transient molecular species that exist during chemical reactions. Perhaps the most widely studied transition-state mimics are those for phosphoryl-transfer reactions, particularly intermediates in S_N_2-type nucleophilic attack on phosphates that leads to new nucleophile–phosphorus bonds with the breakage of phosphorus–oxygen bonds. The transition state for S_N_2 attack on phosphate is a trigonal bipyramidal arrangement of atoms around the central phosphate, where the attacking nucleophile and the leaving-group atom occupy the apical positions and the nonbridging O atoms of the phosphate occupy the equatorial positions. Planar ions such as nitrate (NO_3_^−^) and tetrafluoroaluminate (AlF_4_^−^) can provide informative transition-state mimics because they are effective steric and electronic mimics of the planar PO_3_ moiety of the transferred phosphate in the reaction. However, the most often utilized and perhaps the best transition-state mimic is vanadate, a reactive phosphate mimic which, in addition to adopting a trigonal bipyramidal geometry in many phosphoryl-transfer enzyme active sites, readily forms long (∼2 Å) bonds along its apical positions. Here, we also present two vanadate-bound structures of *Burkholderia* PGAM that provide the most accurate transition-state analog structures for this class of enzymes to date.

Beyond the structures of substrates, products and transition-state mimics, which can provide insight into reaction mechanisms, fragment-bound structures of enzymes can provide additional chemical starting points for small-molecule inhibitor design. Fragment-based drug-discovery methods (reviewed in Rees *et al.*, 2004 ▶; Congreve *et al.*, 2008 ▶) are focused on the discovery of small molecules that bind to specific sites on macromolecular targets. An ensemble of fragment-bound structures can be used to map binding sites and assist in structure-based drug-design efforts. Here, we present two examples of fragment-bound structures: one from a metabolite-based fragment collection called Fragments of Life (Davies *et al.*, 2009 ▶) and one from an alternate crystal form grown in malonate. Taken together, the ensemble of X-ray crystal structures of *B. pseudomallei* PGAM presented here provides one of the most complete pictures of a phosphoglycerate mutase-family enzyme and its interactions with small molecules elucidated to date.

## 2. Methods

### 2.1. Protein expression and purification

Full-length 2,3-bisphosphoglycerate-dependent phosphoglycerate mutase (accession ID YP_332076) was amplified from purified *B. pseudomallei* strain 1710b genomic DNA (kindly provided by Rajinder Kaul, University of Washington) using the following primer sequences: FWD primer 5′-CTCACCACCACCACCACCATATGT­ACAAGCTCGTTCTCATCCG-3′ and REV primer 5′-ATCCTAT­CTTACTCACTTATGCCGCGGACTTGCCCTGCT-3′ (Invitrogen). The PCR conditions were optimized for the high G+C content of *Burkholderia* DNA. Each 50 µl reaction consisted of 27.1 µl dH_2_O, 10 µl Phusion GC Buffer (5×) with 7.5 m*M* MgCl_2_ (NEB, catalog No. F-519), 0.4 µl 25 m*M* dNTPs (Qiagen, catalog No. 201912), 2 µl DMSO, 0.5 µl (1 U) Phusion High-Fidelity DNA polymerase (NEB, catalog No. F-530L), 4 µl FWD primer, 4 µl REV primer and 2 µl (20 ng) *B. pseudomallei* 1710b genomic DNA; thermal cycling con­ditions were 361 K for 30 s, followed by 30 cycles of 361 K (15 s), 338 K (15 s) and 345 K (30 s) and a final extension of 345 K for 3 min (Bio-Rad, MJ Research PTC-200 thermal cycler). PCR amplicons were run on agarose gel to verify the expected size of the amplified gene; the band was then excised from the gel and extracted from the agarose using a QiaQuick kit (Qiagen, catalog No. 28181). The purified PCR product was cloned into the expression vector BG1861, which expresses the protein product fused to a minimal noncleavable polyhistidine tag (MAHHHHHH) on the N-terminus, by ligation-independent cloning (LIC; Aslanidis & de Jong, 1990 ▶). Briefly, the purified PCR product was treated with T4 DNA polymerase in the presence of the single nucleotide dCTP, creating overhangs arising from the 3′-to-5′ specific exonuclease activity, and then annealed with compatible, linearized and T4-treated BG1861 vector (Mehlin *et al.*, 2006 ▶). Annealed vector and insert were transformed into NovaBlue competent cells (Novagen, catalog No. 71011-4) and plated on LB agar (BD Difco LB Agar, Miller; BD, catalog No. 244520) with 50 µg ml^−1^ each of ampicillin (Anatrace, catalog No. A1000) and carbenicillin (Duchefa Biochemie, catalog No. C0109.0025) to select for cells carrying the expression plasmid. The presence of the insert was verified by colony PCR (using the above conditions, but the colony was resuspended in water and used as template instead of purified DNA). Plasmid DNA was purified (QIAprep Turbo Miniprep kit; Qiagen, catalog No. 27191) from 1 ml overnight cultures and then transformed into the expression host *E. coli* BL21(DE3)-R3-pRARE2 cells (a gift from SGC Toronto, Toronto, Ontario) by heat-shock methods.

Starter cultures of 3 ml LB medium with appropriate antibiotics were grown for ∼18 h at 310 K. ZYP-5052 auto-induction media was prepared fresh as per Studier’s protocols (Studier, 2005 ▶). Antibiotics were added to 2 l bottles of sterile auto-induction medium. The bottles were inoculated with all of the overnight culture and then grown for 72 h at 293 K in a LEX bioreactor. After 72 h growth, the cell paste was pelleted by centrifugation at 4000*g* for 20 min at 277 K and the resulting cell pellet was frozen in liquid nitrogen and stored at 193 K.

To prepare the protein, the cell paste was solubilized in lysis buffer (25 m*M* HEPES, 500 m*M* NaCl, 5% glycerol, 30 m*M* imidazole, 0.5% CHAPS, 10 m*M* MgCl_2_, 1 m*M* TCEP, 250 ng ml^−1^ AEBSF pH 7.0) with 0.01 g lysozyme added and sonicated for 30 min. After sonication, samples were treated with Benzonase and then centrifuged for 1 h to clarify the cell debris. The protein was purified by immobilized metal-ion affinity chromatography on nickel Sepharose columns with equilibration buffer (25 m*M* HEPES pH 7.0, 500 m*M* NaCl, 5% glycerol, 30 m*M* imidazole, 1 m*M* TCEP and 0.025% azide) using an ÄTKAexplorer. After thorough washing, bound protein was eluted off the nickel column by addition of elution buffer (25 m*M* HEPES pH 7.0, 500 m*M* NaCl, 5% glycerol, 1 m*M* TCEP, 250 m*M* imidazole and 0.025% azide). Fractions from the nickel column were analyzed for protein content and pooled. Peak fractions of protein were further purified using a HiLoad 26/60 Superdex size-exclusion chromatography (SEC) column on an ÄTKAprime at 1 ml min^−1^ with a running buffer consisting of 25 m*M* HEPES pH 7.0, 500 m*M* NaCl, 5%(*v*/*v*) glycerol, 0.025%(*w*/*v*) sodium azide and 2 m*M* DTT. SEC fractions were analyzed by SDS–PAGE and the purest fractions were pooled and concentrated in the SEC buffer to a final concentration of 26 mg ml^−1^. Protein samples were aliquoted into 100 µl volumes, flash-cooled in liquid nitrogen and stored at 193 K.

### 2.2. Crystallization

Crystals were grown by sitting-drop vapour diffusion by mixing 0.4 µl reservoir solution with 0.4 µl protein solution over a reservoir volume of 80 µl in a 96-well Compact Jr plate (Emerald BioSystems). For PDB entries 3ezn, 3gp3, 3gp5, 3gw8 and 3fdz, crystals were harvested from a single drop in the primary crystallization screen set up at 289 K. The reservoir was condition B4 of the Cryo screen (Emerald BioSystems) and consisted of 5% PEG 1000, 10% glycerol, 30% PEG 600 and 100 m*M* MES pH 7.5. The drop contained dozens of plate-like crystals smaller than 50 µm in size. A single crystal was harvested with a Litho Loop and flash-cooled for data collection by plunging the loop directly into liquid nitrogen. Following data collection and solution of the unliganded structure, four additional ligand-bound structures were obtained by soaking. The 2-phosphoserine and 2,3-bisphosphoglycerate/3-phosphoglycerate structures were obtained *via* overnight soaks in crystallization reservoir solution supplemented with 20 m*M* 2-phosphoserine or 20 m*M* 3-phospho­glycerate, respectively. The vanadate–glycerol structure was obtained *via* a 2 h soak in crystallization reservoir solution supplemented with 2 m*M* activated sodium orthovanadate. Activated vanadate was prepared following the method of Gordon (1991 ▶). Briefly, sodium orthovanadate was dissolved in water, adjusted to pH > 10.0 with concentrated sodium hydroxide and heated at 368 K for ∼5 min. The process of adding sodium hydroxide and heating was repeated until the pH remained above 10 and the solution remained clear and colorless upon cooling. The vanadate–3-phosphoglycerate structure was obtained *via* a 3 h soak in crystallization reservoir solution supplemented with 2 m*M* activated sodium orthovanadate and 20 m*M* 3-phosphoglycerate. Structure 3lnt was solved from a crystal grown by sitting-drop vapour diffusion as described above but was obtained from condition C3 of the Index HT screen (Hampton Research), with a reservoir solution consisting of 2.4 *M* sodium malonate pH 7.0. A single crystal was transferred to a cryoprotectant consisting of 2.4 *M* sodium malonate pH 7.0 plus 25%(*v*/*v*) ethylene glycol prior to flash-cooling for data collection.

### 2.3. Data collection and structure determination

Data for structures 3ezn, 3gp5 and 3fdz were collected using a Rigaku MicroMax-007 HF rotating-anode X-ray source with VariMax Cu-HF optics and a Saturn 944 CCD detector. Data for structures 3gw8 and 3lnt were collected using a Rigaku SuperBright FR-E+ X-­ray generator with Osmic VariMax HF optics and a Saturn 944+ CCD detector. Data for structure 3gp3 were collected on beamline 23-ID-D of the Advanced Photon Source in Argonne, Illinois, USA. Surprisingly, this crystal was not isomorphous to the other four from the same drop and its triclinic unit cell was almost twice the size of the others. The data sets were reduced and scaled using the *HKL*-2000 software suite (Otwinowski & Minor, 1997 ▶), except for structure 3lnt, which was processed using *XDS* (Kabsch, 2010 ▶).

The unliganded structure of *B. pseudomallei* PGAM was solved by molecular replacement using *Phaser* from the *CCP*4 software suite (Winn *et al.*, 2011 ▶) with the C-terminal domain (residues 129–336) of the *Chlorobium tepidum* BchU protein (PDB entry 1x19; Wada *et al.*, 2006 ▶) as the search model, with non-identical side chains truncated by the program *CHAINSAW*. Molecular replacement was successful, despite the low sequence identity (∼19%) between the search model and the target. The structure was initially rebuilt with *ARP*/*wARP*, followed by numerous reiterative rounds of refinement in *REFMAC* and manual building using the *Crystallographic Object-Oriented Toolkit* (*Coot*; Emsley & Cowtan, 2004 ▶). Subsequent structures were solved by molecular replacement using the protein-only model of 3ezn as the search coordinates. Ligand-bound models were built by alternating rounds of refinement with *REFMAC* and manual building and inspection in *Coot*. Data-collection and refinement statistics are given in Tables 1 ▶ and 2 ▶.

## 3. Results and discussion

### 3.1. Ensemble of *B. pseudomallei* structures

The first of the *B. pseudomallei* PGAM structures to be solved was 3ezn, the ‘apo’ structure without ligands (the only nonprotein atoms in the model other than solvent waters are part of polyethylene glycol molecules on the surface of the proteins). The apo structure con­tained a dimer in the asymmetric unit and the active site of each protein monomer was unencumbered by crystal-packing interactions, contained a few ordered solvent water molecules and showed no evidence of phosphorylation of the active-site residue His9. Four of the five ligand-bound structures that followed (PDB entries 3fdz, 3gp3, 3gp5 and 3gw8) were solved from soaked crystals harvested from the exact same 0.8 µl sitting-drop vapor-diffusion well that yielded the apo structure. Thus, the unliganded structure is an excellent control which represents the initial state of the enzyme in the crystal form prior to the addition of ligands.

**Figure 1:**
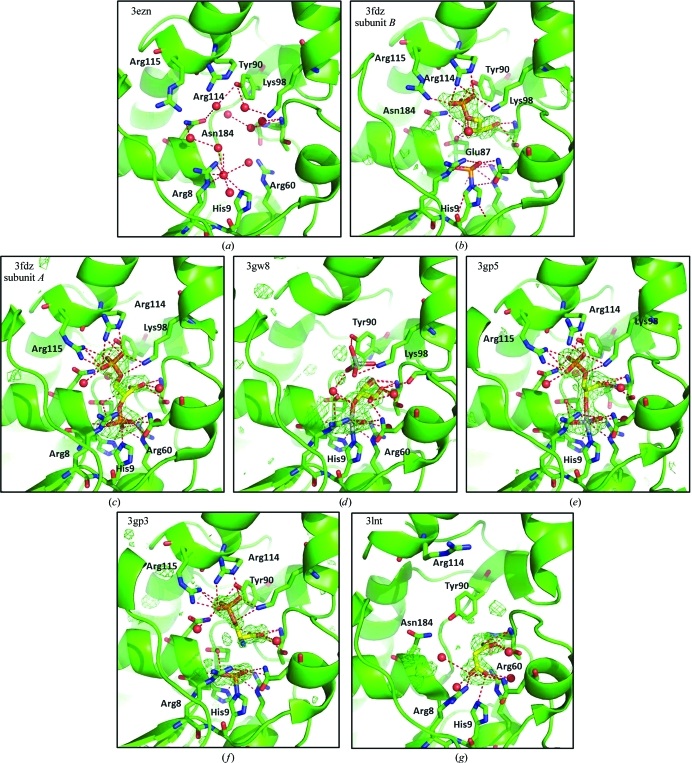
Ensemble of *B. pseudomallei* PGAM structures. Protein models are displayed in green as ribbon structures, with key residues rendered as stick structures. Ligands are shown in stick representation with C atoms colored yellow. Wire mesh represents the 3.0σ level of the (*F*_o_ − *F*_c_) OMIT map calculated for the final structure with ligands removed. Polar contacts are rendered as red dashed lines. All ligands were modelled at 100% occupancy, with the exception of the tetrahedral vanadate in 3gw8 (50%) and the two alternate conformations of glycerol in 3gw8 (50% each). In all cases occupancies were held constant and not refined. (*a*) PDB entry 3ezn, apo structure; (*b*) PDB entry 3fdz subunit *B*, bound 3-phosphoglycerate with phosphohistidine; (*c*) PDB entry 3fdz subunit *A*, bound 2,3-bisphosphoglycerate; (*d*) PDB entry 3gw8, vanadate–glycerol complex; (*e*) PDB entry 3gp5, vanadate–3-phosphoglycerate complex; (*f*) PDB entry 3gp3, bound 2-phosphoserine; (*g*) PDB entry 3lnt, malonate-bound structure.

Structure 3fdz was obtained *via* an overnight soak of a single crystal in crystallization solution supplemented with 20 m*M* 3-phosphoglycerate. Unexpectedly, the resultant crystal structure exhibited electron density consistent with the presence of 2,3-bisphospho­glycerate in one of the monomers and 3-phosphoglycerate along with phosphorylation at His9 in the other. These observations strongly suggest that PGAM is active in the *P*1 crystal form and that the 2,3-bisphosphoglycerate intermediate was generated by enzymatic con­version of 3-phosphoglycerate. The presence of two distinct stages of the PGAM catalytic cycle in the same crystal structure is a serendipitous discovery, but difficult to rationalize in light of the observed structure. The two monomers in the asymmetric unit are related by noncrystallographic symmetry and thus are not identical, providing distinct chemical and structural environments for each active site. However, least-squares superposition of the two monomers (as implemented in *Coot*; Emsley & Cowtan, 2004 ▶) revealed an r.m.s. deviation of 0.70 for the apo structure and of 0.63 for the 3-phosphoglycerate-soaked structure. Closer inspection shows that the active-site regions are even more similar, with all ligand-contacting amino-acid side chains except Arg115 adopting nearly identical conformations (Fig. 1 ▶).

The ensemble of *Burkholderia* PGAM structures also includes two fragment-bound models. The term ‘fragments’ is used in the context of fragment-based drug discovery, where a fragment is any small molecule of molecular weight approximately less than 300 Da. Fragment-screening efforts can often lead to the identification of small molecules that bind to protein targets with relatively low affinity but with high specificity. The utility of fragments for structure-based inhibitor design is that they allow researchers to probe protein binding pockets for sites capable of binding different chemical groups, which may subsequently be linked through chemical elaboration to create larger high-affinity inhibitors. The structures of PGAM bound to 2-phosphoserine and malonate (PDB entries 3gp3 and 3lnt, respectively) are examples of nonsubstrate fragment molecules that may inform inhibitor design.

The small molecule 2-l-phosphoserine was selected from the Fragments of Life library (Emerald BioStructures) based on its structural similarity to 3-d-phosphoglycerate. Both 2-phosphoserine and 3-­phosphoglycerate share a secondary carboxylate group and a phosphate linkage. The key differences between the two small molecules are that they have inverted chirality at the C2 atom and serine has an amino group in place of a hydroxyl. PGAM tolerates binding of this nonsubstrate molecule, probably owing to the nearly unchanged relative positions of the phosphate and carboxylate moieties, which are highly coordinated by the active-site residues Thr21, Gly22, Tyr90, Lys98, Arg114 and Arg115. The replacement of the secondary hydroxyl by a secondary amine is expected to make 2-­phosphoserine unreactive with PGAM and the fragment is expected to be an inhibitor. Also of note in the 2-phosphoserine structure is the unusual structure of the phosphorylated histidine residue His9. The bond angles between the phosphate O atoms, the central P atom and His9 N^∊2^ are close to 90° and the P—N^∊2^ distance is long for a nitrogen–phosphorus bond at ∼2 Å. This geometry is reminiscent of the planar arrangement around phosphate observed in glucose 1-phosphate-bound β-phosphoglucomutase (Lahiri *et al.*, 2003 ▶; PDB entry 1o08). The β-phosphoglucomutase structure was interpreted as a true transition state for phosphate transfer captured in a crystal structure. Unlike the β-phosphoglucomutase structure, where there is an ∼2 Å distance between the P atom and both the enzyme nucleophile and the substrate leaving group, there is a very long (>3.8 Å) distance between the P atom and the nearest atom of the 2-phosphoserine.

The second fragment-bound structure (PDB entry 3lnt) contains malonic acid bound in the active site. The dicarboxylic acid adopts a bound conformation that places one carboxylate group in the carboxylate-binding pocket formed by Thr21 and Gly22, while the other carboxylate group is oriented towards His9 near the positively charged region responsible for binding the transferred phosphate. This structure was obtained from a different crystal form that was grown from 2.4 *M* sodium malonate. The alternate crystal packing in the malonate-grown crystals coincided with a significant shift of the α-­helix containing residues Arg114 and Arg115 relative to its position in the *P*1 crystal form. Arg114 and Arg115 are largely responsible for binding the 3-phosphate of the substrate molecule and it is unknown whether the malonate-grown crystal form could accommodate 3-­phosphoglycerate binding. While useful for elucidating the binding mode of a dicarboxylic acid fragment, this crystal form is completely unsuitable for soaking of additional ligands since the crystallant itself is also the ligand.

### 3.2. Vanadate structures

Vanadate is a reactive metal oxoanion that is capable of adopting structures that mimic the transition state of phosphoryl-transfer reactions (Lindquist *et al.*, 1973 ▶; VanEtten *et al.*, 1974 ▶). Vanadate has been successfully used to capture transition-state mimics in X-ray crystal structures of a variety of enzymes that perform chemistry on a phosphate group. A survey of the crystallographic literature in 2004 (Davies & Hol, 2004 ▶) revealed vanadate to be a versatile molecular tool that had elucidated transition-state structures for enzymes in four of the six EC categories of enzymes from crystals grown at pH 5.0–7.8. Based on this history, we reasoned that our crystals of *Burkholderia* PGAM (at pH 7.5) were excellent candidates for soaking with vanadate compounds.

**Figure 2:**
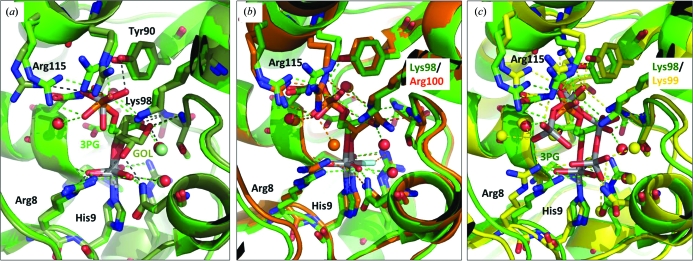
Comparison of vanadate- and AlF_4_^−^-bound structures of phosphoglycerate mutase enzymes. Pairwise alignments were performed using the *SSM* superpose function from the *CCP*4 program suite. (*a*) Superposition of the structures from PDB entries 3gw8 (vanadate–glycerol, pale green) and 3gp5 (vanadate–3-phosphoglycerate, bright green). (*b*) Superposition of the structures from PDB entries 3gp5 (vanadate–3-phosphoglycerate, green) and 2f90 (human erythrocyte BPGM with bound tetrafluoroaluminate and 3-­phosphoglycerate, orange). (*c*) Superposition of the structures from PDB entries 3gp5 (vanadate–3-phosphoglycerate, green) and 1e59 (*E. coli* PGAM with bound tetravanadate, yellow).

*E. coli* PGAM has previously been subjected to crystallographic investigation with vanadate (Bond *et al.*, 2002 ▶; PDB entry 1e59). However, rather than binding in a trigonal bipyramidal configuration at the nucleophilic histidine, a polymer of four tetrahedral vanadate moieties was visualized in the substrate-binding site. The binding of tetravanadate rather than a monomeric transition-state mimic vanadate species in the *E. coli* PGAM structure was likely to be a result of the methods employed for crystal growth. The published crystallization conditions listed the use of 100 m*M* NaVO_3_ rather than ‘activated’ Na_3_VO_4_ and described the slow development of a yellow color consistent with vanadate polymerization during the period of crystal growth. Bond and coworkers hypothesized that the tetravanadate species visualized in their structure represented not a transition-state mimic but rather provided the locations of preferred binding sites for carboxylate and phosphate anions. This hypothesis is strengthened by comparison of the tetravanadate structure with the *Burkholderia* vanadate-bound structures, which are proper transition-state mimics (Fig. 2*c* ▶).

Two vanadate structures with *Burkholderia* PGAM were obtained, each with additional molecular entities covalently bound to the vanadate. One of the properties that makes vanadate such a powerful transition-state mimic is its ability to exchange its O atoms and derivatize with a variety of ligands. Low-order covalent bonds can be formed between the V atom and the apical ligands of the trigonal bipyramidal configuration representing the attacking nucleophile and leaving group. Derivitization of vanadate by glycerol is not without precedent; in a crystal structure of human tyrosyl DNA phosphodiesterase, glycerol from the cryoprotectant became covalently bound to vanadate at the active site at one apical and one equatorial position (Davies *et al.*, 2002 ▶). Glycerol was present in the crystallization and soaking solutions of PGAM at 10%(*v*/*v*) concentration and its visualization in the crystal structure (PDB entry 3gw8) was not unexpected. The crystal structure revealed that the glycerol O2 atom had become the apical ligand of vanadate opposite the N^∊2 ^atom of His9. In this arrangement, glycerol adopts a conformation and position similar to the glycerate moiety of the natural PGAM substrate. Interestingly, electron density consistent with a tetrahedral oxoanion, presumed to be VO_4_, was observed adjacent to the bound glycerol, occupying the position of the binding site for the 3-phosphate of the substrate. Difference electron density was weaker for the tetrahedral vanadate, but the presence of vanadium was confirmed by calculation of an anomalous difference map (data not shown). The tetrahedral vanadate oxoanions were modelled at 50% occupancy.

An even more accurate transition-state mimic was achieved in structure 3gp5. In this structure, 20 m*M* 3-phosphoglycerate was present in the soaking solution in addition to vanadate. The resultant structure was comprised of vanadate in a trigonal bipyramidal con­figuration, with the N^∊2^ atom of His9 and the O2 atom of 3-phosphoglycerate contributing apical ligands. The three equatorial atoms of vanadate were approximately coplanar and formed polar contacts with the side chains of Arg8, Asn15, Arg60, Glu87 and His182 and the backbone N atom of Gly183. This arrangement of the equatorial O atoms is nearly identical in the glycerol-bound structure, indicating that the vanadate is providing an accurate structure of the transition-state structure of phosphate despite the presence of a nonsubstrate ligand such as glycerol. The only similar example of a transition-state mimic for a phosphoglycerate mutase-family enzyme in the PDB is a structure of human erythrocyte bisphospho­glycerate mutase with tetrafluoroaluminate and 3-phosphoglycerate (Wang *et al.*, 2006 ▶; PDB entry 2f90). In this structure, the square planar AlF_4_^−^ ion occupies nearly the same relative position as vanadate. When compared with the vanadate structures, the interatomic distances from the central Al atom are similar to the interatomic distances around vanadium. However, tetrafluoroaluminate is a less accurate transition-state mimic because it has four equatorial F atoms instead of three equatorial O atoms as found in vanadate.

Together with the 3-phosphoglycerate-bound and 2,3-bisphospho­glycerate-bound structures, the vanadate–3-phosphoglycerate structure provides a complete picture of the first stage of the PGAM reaction proceeding from phosphoenzyme plus substrate to transition state to enzyme plus intermediate product.

## 4. Conclusions

The ensemble of six X-ray crystal structures of *B. pseudomallei* PGAM presented here provides for the first time a comprehensive view of a phosphoglycerate mutase enzyme complete with bound ligands encompassing substrate, intermediate, transition-state mimic and fragment-bound structures. Taken together, these crystal structures allow the definitive identification of key amino-acid residues involved in substrate binding and catalysis. The inclusion of two metabolite-like fragment structures in the ensemble (2-phosphoserine and malonate) provides starting points for elucidating ligand-binding flexibility and potential for small-molecule inhibitor design.

## Supplementary Material

PDB reference: 2,3-bisphosphoglycerate-dependent phosphoglycerate mutase, 3ezn

PDB reference: 3fdz

PDB reference: 3gp3

PDB reference: 3gp5

PDB reference: 3gw8

PDB reference: 3lnt

## Figures and Tables

**Table 1:** Data-collection statistics Values in parentheses are for the highest of 20 resolution shells.

Ligand	Apo	2-Phosphoserine	Vanadate + 3-phosphoglycerate	Vanadate (+ glycerol)	2,3-Bisphosphoglycerate + 3-phosphoglycerate	Malonate
Space group	*P*1	*P*1	*P*1	*P*1	*P*1	*P*6_5_
Unit-cell parameters						
*a* (Å)	44.94	49.26	44.75	45.18	44.39	119.62
*b* (Å)	49.08	72.05	48.98	49.11	48.48	119.62
*c* (Å)	62.11	78.04	62.96	62.59	62.09	103.67
α (°)	107.11	107.96	105.46	106.42	106.03	90.0
β (°)	91.19	93.00	91.15	91.18	91.54	90.0
γ (°)	107.81	104.20	107.42	107.61	107.50	120.0
Wavelength (Å)	1.5418	0.97934	1.5418	1.5418	1.5418	1.5418
Resolution range (Å)	50.0–2.10 (2.15–2.10)	50.0–1.50 (1.54–1.50)	50.0–2.25 (2.33–2.25)	50.0–1.93 (1.98–1.93)	50.0–2.25 (2.31–2.25)	50.0–2.10 (2.15–2.10)
Unique reflections	26261	149575	21960	34058	20934	49080
Multiplicity	3.7 (3.6)	3.1 (2.1)	2.0 (1.9)	3.4 (2.20)	3.4 (3.0)	7.4 (7.0)
Completeness (%)	93.7 (83.2)	95.3 (84.9)	94.5 (82.1)	92.8 (80.4)	93.4 (90.5)	99.9 (99.9)
*R*_merge_† (%)	4.1 (13.7)	8.1 (59.8)	6.80 (24.5)	6.60 (30.1)	7.1 (34.5)	8.50 (72.1)
Mean *I*/σ(*I*)	25.7 (9.9)	18.1 (1.4)	13.1 (3.45)	22.0 (2.89)	11.43 (2.60)	18.8 (2.30)

† *R*_merge_ = 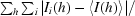 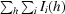.

**Table 2:** Refinement and model statistics Values in parentheses are for the highest of 20 resolution shells.

Ligand	Apo	2-Phosphoserine	Vanadate + 3-phosphoglycerate	Vanadate (+ glycerol)	2,3-Bisphosphoglycerate + 3-phosphoglycerate	Malonate
Resolution range (Å)	50.0–2.10 (2.15–2.10)	50.0–1.50 (1.54–1.50)	50.0–2.25 (2.33–2.25)	50.0–1.93 (1.98–1.93)	50.0–2.25 (2.31–2.25)	50.0–2.10 (2.15–2.10)
*R*_cryst_†	0.150 (0.147)	0.237 (0.429)	0.171 (0.215)	0.187 (0.260)	0.183 (0.252)	0.170 (0.222)
*R*_free_†	0.204 (0.227)	0.268 (0.425)	0.234 (0.316)	0.227 (0.308)	0.256 (0.331)	0.196 (0.251)
R.m.s.d. bonds (Å)	0.020	0.027	0.019	0. 009	0.013	0.015
R.m.s.d. angles (°)	1.650	2.114	1.754	1. 200	1.526	1.385
Protein atoms	3726	7424	3771	3701	3668	3808
Nonprotein atoms	402	850	298	409	263	283
Mean *B* factor (Å^2^)	14.88	19.20	22.90	22.54	23.70	27.65
Ligand *B* factor‡ (Å^2^)	N/A	23.32	19.66	31.04	30.00	33.57
Residues in favored region (%)	83.7	85.8	83.3	87.2	82.1	83.9
Residues in allowed region (%)	98.7	98.9	98.9	98.7	99.3	99.2
*MolProbity* score [percentile]	1.90 [86th]	1.69 [70th]	1.78 [95th]	1.31 [99th]	2.01 [87th]	1.18 [100th]
PDB code	3ezn	3gp3	3gp5	3gw8	3fdz	3lnt

† *R*_cryst_ = 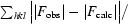 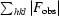. The free *R* factor was calculated using 5% of the reflections omitted from the refinement (Winn *et al.*, 2011 ▶).

‡ Ligand *B* factors are for ligands in the active sites of the protein monomers at 100% occupancy., Ligands from solvent (PEG, glycerol *etc*.) were not included in the calculation of these *B* factors.
